# Discovery, reusability, sustainability: Exploring principles for web-map application development with *Peripleo*

**DOI:** 10.1007/s42803-025-00112-w

**Published:** 2025-12-15

**Authors:** Gethin Rees, Leif Isaksen, Stephen Gadd, Rainer Simon

**Affiliations:** 1 King’s College London, London, UK; 2 University of Exeter, Exeter, UK; 3 School of Advanced Study, London, UK; 4 Cultural Heritage, Web Maps, Geography, Linked Open Data, Historic Environment, Museum, Library, Vienna, Austria

## Peripleo and the Locating a National Collection project

Maps have been used to present cultural heritage on the web since the 1990 s (Leusen et al., 1996, p. 513). In 2025 it is commonplace for cultural heritage organisations and digital humanities projects to create bespoke web-map applications or use platforms such as Google’s *MyMaps* to make collections available. Indeed, since 2012, more than one third of nominations in the Digital Humanities (DH) awards ‘Best DH Data Visualization’ category have been interactive maps (Cummings, 2022). Web maps offer a method of visualisation that is familiar to academic researchers and the public more widely from using applications such as Google or Apple maps. However, the ease with which these maps can be deployed has at times led to a lack of attention to their purpose, users and longevity. This article asks, how can the needs of stakeholders inform the design of web maps? Research by the Locating a National Collection (LaNC) project examined the skills, requirements and motivations of three groups: cultural heritage organisations, cultural heritage professionals and end-users in the form of public audiences. Quantitative surveys, interviews, focus groups and structured assessment of existing web-map interfaces led to the definition of three key principles for application development: sustainability, reusability and discovery (Rees et al., 2022a; Rees et al., 2022b). LaNC then explored the implementation of these principles in developing the *Peripleo* web-map application (Simon et al., 2022). Created by Rainer Simon from the Austrian Institute of Technology, with assistance from Stephen Gadd, Gethin Rees, Leif Isaksen and Victoria Morris, *Peripleo* is specifically designed to help cultural heritage professionals to present collections to the public. Despite being a prototype, the application is open-source and documented, making it available to anyone to deploy. LaNC researcher Stephen Gadd saw a need for a curation tool to create datasets for *Peripleo* and developed a prototype which he named *Locolligo* (Gadd, 2023). Collectively, this software demonstrates how a linked open data approach can be applied to connect and visualise collections from across the UK’s cultural heritage sector (Kahn et al., 2021).

**Fig. 1:**
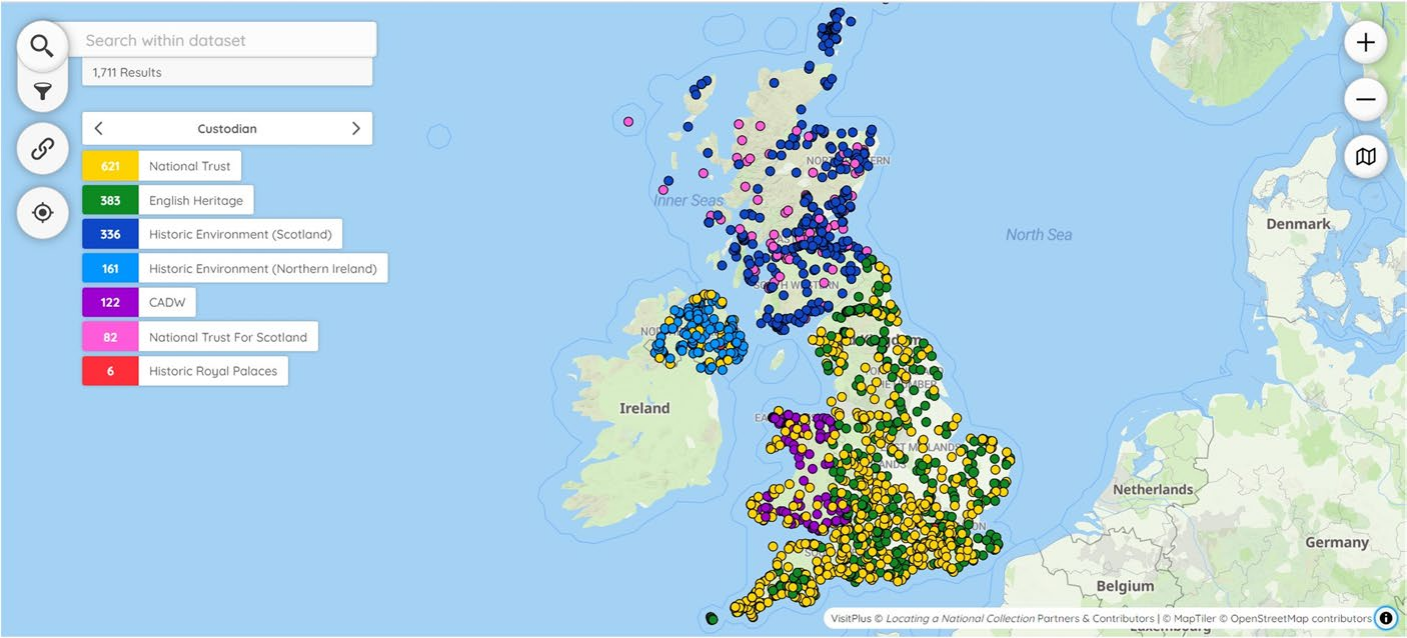
*Peripleo* web-map interface displaying VisitPlus dataset (Gadd & Rees, 2022)

At the outset of LaNC, the investigative team were open-minded regarding the technologies and features to implement in *Peripleo*. This article outlines the design choices that the investigative team made in the application’s development and how they were influenced by the requirements of the UK’s cultural heritage sector. The article’s research objective is to provide a rationale for the development of three elements of web maps: architecture, dataset and user interface. This rationale consists of a set of principles developed over the course of stakeholder research. These principles were developed not only in relation to end-users but also by drawing on the capabilities and motivations of those who would create instances of Peripleo, cultural heritage communities and organisations. The principles of discovery, reusability and sustainability have potential applications beyond web maps. Therefore, a second research objective is to offer a pathway for implementing stakeholder research in the design and development of software applications. First and foremost, *Peripleo* was designed to enable end-users to explore and find web pages through their visual representation in an act of ‘discovery’. In connecting cultural heritage records from across different collections and guiding end-users to click through to web pages, the aim is to assist researchers in ‘the activity of seeking out objects of research, research results, or other information’ (TaDiRAH, n.d.). Described as ‘discovery’ in the Taxonomy of Digital Research Activities in the Humanities (TaDiRAH), the activity is usually associated with tasks like querying, searching or browsing. Second, the aim in developing *Peripleo* was to lower the barriers to creating maps of linked open data and to make these maps openly accessible, described as ‘reusability’ in the foregoing discussion. A reusable application is designed with the intention of making it as simple as possible for cultural heritage professionals to make an instance of *Peripleo* —their own version presenting their own data— publicly available on the web. Such professionals will be described as ‘creators’ in the remainder of the article. They work within cultural heritage organisations, have varying levels of digital skill but typically cannot write code. Ensuring that creators can work with datasets and integrate these within *Peripleo* is as important to reusability as providing server architecture that is straight-forward to host and maintain. Given the initial investment of effort, instances of *Peripleo* must be sustainable and remain available for a considerable period. Sustainability rests as much on finances as on technical considerations. Funding is usually scarce and time-limited so it was resolved to try and keep the labour and costs involved in web hosting and maintenance to a minimum. To this end, *Peripleo* adopts a front-end only design that eschews the need for a dedicated server-side application and database, minimising overheads like setup and maintenance. This low-cost and comparatively straightforward route to hosting can expedite reuse of the application in the cultural heritage sector. The article’s contribution is to build bridges between stakeholders and application development, to understand how the capabilities and motivations of those using and creating instances of applications can inform design decisions and support adoption. The findings can inform paradigms such as the FAIR principles (Findability, Accessibility, Interoperability, Reusability) (Barker et al., 2022), as well as user-centred (Still & Crane, 2017) and minimal-computing (Risam & Gil, 2022) approaches to application development (Fig. 1).

## Stakeholder research

### Project background and development rationale

LaNC helped cultural heritage organisations to use location data —such as where objects were made and used or the places they depict and describe— to connect cultural heritage collections from across the UK. Funded by the Towards a National Collection programme, a major investment in research by the UK’s Arts and Humanities Research Council, LaNC’s research explored how geographical information offers a particularly effective way to address two of the programme's aims. First, to ‘dissolve barriers between different collections’ and second, to foster ‘wider and better-informed public engagement’ (Rees et al., 2022a). To address these aims, LaNC drew on methods and tools developed by the Pelagios Network, a long-running initiative that has formed a ‘community of practice’ who work with linked open data through common references to places, people and time periods (Kahn et al., 2021). LaNC applied these methods to connect the collections of two types of cultural heritage organisation who acted as project partners. The first are custodians of the historic environment who manage and record heritage sites for the purpose of conservation, research and visitors: Historic Environment Scotland, Historic England, English Heritage, National Trust and Historic Royal Palaces. The second are galleries, libraries, archives, museums (GLAMs) whose collections encompass material objects, namely the British Library and the Portable Antiquities Scheme. The focus here is on digital representations of both types of cultural heritage organisations’ collections that take the form of web pages. Termed ‘records’ in the remainder of the article, they include representations of objects, documents, monuments or buildings, for example, and contain digitised or born-digital content like images or sounds, structured data, text or Uniform Resource Locators (URLs). The LaNC team then went on to build a web-map application to make these connected records available for discovery. This article focuses on the development process of this software. The traditional or waterfall approach breaks this process down into three phases: first, an initial phase of gathering requirements and planning; second, a development phase of design, development and implementation; and third, a final phase of testing (Reiff & Schlegel, 2022, p. 49). The approach to developing Peripleo could be described as hybrid, however, as work on the user interface was iterative. Testing fed back into development, meaning elements of an agile approach were adopted (Reiff & Schlegel, 2022, p. 46). The three principles facilitated the implementation of requirements and planning from the initial phase in the later development phase. Thus, principles act as a bridge between gathering requirements from stakeholders and other web-map interfaces and the development of Peripleo.

### Understanding stakeholders and their requirements

Requirements were first gathered based on the capabilities and motivations of potential creators of instances of Peripleo: cultural heritage communities and organisations. LaNC conducted extensive research with stakeholders to understand the value of their digital collections and different methods for their dissemination. LaNC’s interviews with cultural heritage professionals conducted by Valeria Vitale attested to cultural heritage organisations’ investment in their records, the difficulties in ensuring the public can discover them and a reliance on text-based commercial search engines (Rees et al., 2022a). The work demonstrated that cultural heritage professionals saw value in connecting collections. They felt this would encourage use of the breadth of their organisation’s records and increase visits to web pages, helping end-users to discover records that they might not find otherwise. Whilst cultural heritage professionals were interested in creating web maps of collections there was a gap in the requisite skills. Creators might be highly digitally literate with technologies such as spreadsheets and HyperText Markup Language (HTML) but they are not developers and cannot code. The Towards a National Collection programme is not only interested in building connections between collections but also in using the results to engage the public. LaNC examined the UK public’s values, motivations and behaviours through surveys and focus groups. This audience research, led by Alex Hunt and Valeria Vitale, demonstrated that geography motivates the public to engage with records and drivers such as proximity, travel, genealogy, memory and residence offer powerful connections for diverse audiences. The public’s familiarity and competence with web maps was evident as was their strong desire to use maps that present cultural heritage. The initial phase of gathering requirements from the public has been discussed in detail elsewhere (Rees et al., 2022b).

### Related work in web maps for cultural heritage

**Fig. 2:**
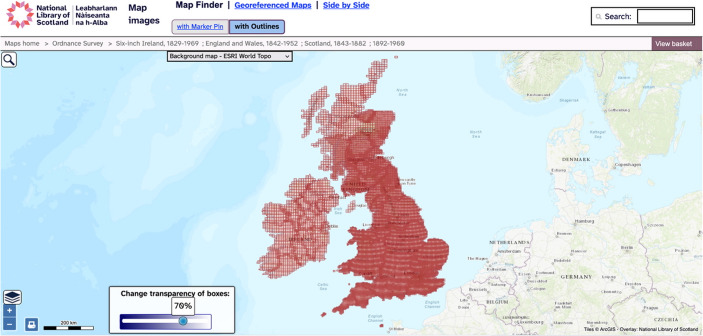
Implementing a web-map component to access cultural heritage, the National Library of Scotland’s Map Finder. (National Library of Scotland, 2023)

The project’s geographical focus and this stakeholder research made building a map interface a priority for LaNC. An older prototype spatio-temporal search and visualisation application also called *Peripleo* had been created by some of the article’s authors to enable end-users to explore objects relating to Greco-Roman antiquity from across 25 digital collections using a web map and filters (Simon et al., 2016). The development process for this older Peripleo offered important insights into the requirements for LaNC’s web map application. Indeed, although the application won the ‘Best DH Data Visualization’ at the DH Awards 2016 (Cummings, 2022), it could be argued that this original *Peripleo* was more influential as a proof of concept than as a research application. The user-interface was designed to help digital humanists explore and discover research objects. The bespoke web-map application had the drawback of being based on a complex server-side architecture that made it difficult to reuse and sustain (Simon et al., 2016, p. 6). The LaNC team saw an opportunity to apply what was learned from this original *Peripleo* in the development of a new web-map interface for the cultural heritage community. LaNC examined the strengths and limitations of existing web-map applications in the UK’s cultural heritage sector through desktop research. Cultural heritage organisations have made extensive use of interactive web maps for the public presentation of cultural heritage records. Such maps typically embed Javascript web-map components, such as *MapLibre* or *Leaflet* (Maplibre, n.d.; Leaflet, n.d.), within HTML pages to create browser-based, interactive maps. The LaNC team examined a selection of these maps through desk-based research, structured interviews with cultural heritage professionals alongside quantitative and qualitative work with public audiences. The work laid out strengths and limitations from the perspective of creators and public end-users. The maps will be divided into two types here: bespoke and platform-based applications (Fig. 2).

**Fig. 3:**
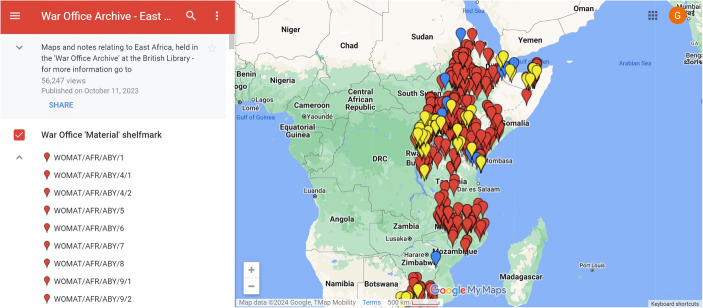
Google’s *MyMaps* platform-based interface displaying ‘Maps and notes relating to East Africa, held in the War Office Archive at the British Library’ (Dykes, 2019)

Heritage sites are the focus of historic environment organisations’ work leading to investment in geospatial data, strong expertise in Geographical Information Systems (GIS) and bespoke web-map applications. Coordinates embedded within historic environment organisations’ records represent sites and expedite visualisation, making maps a mainstay of their websites in the UK. Bespoke web-mapping applications such as Historic Environment Scotland’s *Canmore* (Canmore, n.d.) and Historic England’s *Heritage Gateway* (Heritage Gateway, n.d.) bring together historic-environment data from several sources. Bespoke applications have also been implemented by GLAMs, although less frequently or systematically and usually for specific collections such as the National Library of Scotland’s digitised maps, Legal Deposit of born-digital mapping or iiif records (Ducatteeuw et al. 2025; Fleet, 2019; Rees & Fleet, 2024). Furthermore, bespoke web-mapping applications are widely used by digital humanities projects and also further afield by projects such as Wikimapia that offer opportunities for crowd sourcing geospatial data (Wikmapia, n.d.). Such bespoke applications are tremendously useful resources with several strengths. Their user interfaces are designed specifically for the application in question and they support access to large datasets that do not need to be openly licensed. It is no surprise that they are well used. However, they also have several drawbacks. Bespoke applications typically require complex databases, server-side architecture and considerable investment to develop, implement and sustain. Where open source, it is technically possible for others to take code and reuse this to make their own instances available. However, the need for infrastructure such as servers, alongside associated funds and expertise creates significant barriers to reuse. In contrast to historic environment organisations’ data, geographical information in GLAM metadata or digital text content takes the form of toponyms, postcodes or addresses. Presenting these locations on web maps relies on deriving geospatial data in the form of points, lines or polygons based on coordinates from this text. This extra step makes it difficult for many GLAMs to create maps systematically. Rather, GLAMs, and at times historic environment organisations, have drawn on platforms developed by technology companies including Google’s *MyMaps* on an ad-hoc basis (Google, n.d.) (Fig. 3). For example, the British Library created maps for individual projects for many years using technologies including Google’s *Fusion Tables* (Gonzalez et al., 2010). These platforms aim to help people without expertise in geospatial technologies to create interactive maps. They facilitate the creation of a publicly accessible web map from a simple spreadsheet of coordinates and associated attributes. Platform-based applications are highly reusable for those with a lower level of technical skill which is a considerable strength. Yet reliance on a simple dataset has drawbacks as a single data structure and generic interface must be applied across every use case. Moreover, the use of platforms results in a reliance on technology companies for the continued provision of services leading to problems with sustainability as evidenced by the deprecation of technologies like Adobe’s *Flash Player* and Google’s *Fusion Tables* (Adobe, n.d.; Gonzalez et al., 2010). Bespoke and platform-based web maps offer an innovative window on collections and their implementation in cultural heritage has provided end-users with much enjoyment. Yet the foregoing evaluation has demonstrated their strengths and weaknesses in terms of reusability, sustainability and discovery. Existing applications have strengths in some, but not all, of these areas. In order to meet the requirements of cultural heritage stakeholders, LaNC sought to implement these principles in the development phase of *Peripleo*.

## Web-map application development

### Approach to development

The project team went on to develop the *Peripleo* web-map application alongside several datasets that were used to test the application by building individual instances. The resulting maps are accessible on the web with underlying code and datasets openly available on Github (Simon et al., 2022). The *Peripleo* application is based on three inter-related elements: architecture, dataset and user interface. The remainder of the article examines the development of each of these elements in terms of design, development and implementation (Reiff & Schlegel, 2022, p. 50). The sections that follow explain how the design of each element was informed by the initial planning and requirements phase and the derived principles. This led design decisions to be taken for each of the three elements that would not change. For example, the team decided that the user interface should include filters, search and popup functionality, that the architecture should be front-end only and that the dataset should be based on JSON-LD standards. Following design, development involved writing the code to create the application, followed by implementation, deployment and making the system operational. In the case of the user interface, a third phase of user testing was incorporated in an iterative process that fed the results back into development. The development of the architecture and dataset were not informed by user testing.

Although one interface was built based on a single architecture, the project team created several datasets in order to explore different aspects of the application. The datasets also offered insights into reusability by helping the team to understand the pathway for creators to build instances of *Peripleo*. Decisions on the datasets to create were based on criteria encompassing not only appeal to stakeholders but also technical practicalities. Is it possible to build the dataset from cultural heritage organisations’ data? How do scale, scope and theme affect sustainability, reusability and interface design? An instance of *Peripleo* that LaNC created called VisitPlus will illustrate the approach adopted throughout the article. The VisitPlus dataset unites over 1600 heritage visitor sites of national significance across the whole of the UK such as castles, palaces and prehistoric monuments, managed by Historic Environment Scotland, English Heritage, Cadw, Northern Ireland Department of Communities, National Trust and the National Trust for Scotland with objects from GLAMs and beyond including datasets from the British Library, Portable Antiquities Scheme, ArtUK and the Imperial War Museum (Gadd & Rees, 2022). VisitPlus connects scattered records through common references to places, people and time periods. The intention in bringing these records together was to enhance visits before, during or after being on site. The heritage visitor sites function as a gazetteer. Web pages such as GLAM records are connected to each through common references leading each visitor site to act as a nexus for heritage records. Each of the following sections examines one of three elements of the *Peripleo* application: architecture, dataset and user interface. Using VisitPlus as an example, they describe decisions that the LaNC team took and how they were guided by principles of discovery, reusability and sustainability.

### User interface

The foundation of *Peripleo’s* user interface is the MapLibre Javascript library (Maplibre, n.d.). This library can be included in a web page to produce a web-map component that offers a set of visual features including tiles, vector markers and map projection, and a set of familiar interactive features including zoom and pan. In addition to these core features, several additions were made, including filters, search and an introductory tour to orient the end-user. Many of these features will be familiar to the public from their use of web maps elsewhere meaning the choice of *MapLibre* supported our overall intention to make the interface easy-to-use for a wide public audience. Furthermore, the WebGL technology on which *MapLibre* is based can accommodate tens of thousands of markers and is ideally suited to presenting the breadth of large collections. The MapLibre component was embedded within a user interface that included features such as filters, search and an introductory tour. The development phase for the interface was more complex than for architecture and dataset development, as design, development and implementation were informed by feedback from end-users at different stages. The design process drew on audience research to establish a use case whilst development and implementation drew on user testing to refine the interface’s functionality.

The user interface design process began with the project team developing interface ideas, themes and content based on stakeholder and technical requirements outlined above and the team’s previous experience. The interviews with cultural heritage professionals discussed earlier offered ideas on how the public might engage with collections. Interface ideas portraying different datasets were then worked up into designs to be tested with public focus groups prior to application development using pretotypes. These ‘powerpoint-style’ slides were used to communicate interface functionality to focus-group participants in general terms alongside the types of data it might be possible to access (Savoia, 2011). Pretotypes provide a path for audience research from the initial phase to feed into application development. Their use is discussed in detail in another publication (Rees et al., 2022b). Technical development only took place once this design process was complete.

This work led to the formulation of a discovery use case that the user interface was designed to support. The intention was to guide the end-user towards the goal of discovering a single location that represents a desired record or records and then clicking through to a web page. This process might be repeated but on each occasion the end-user finds a single vector marker and clicks through to a single web page. Achieving this goal rests on three critical affordances of the map:

- A visual overview that displays a breadth of records, presenting options and possibilities for web pages that end-users might not have in mind.
- Connections between several records and the same location to bring diverse collections together.
- A gateway to accessing records, guiding users to click through to web pages.

**Fig. 4:**
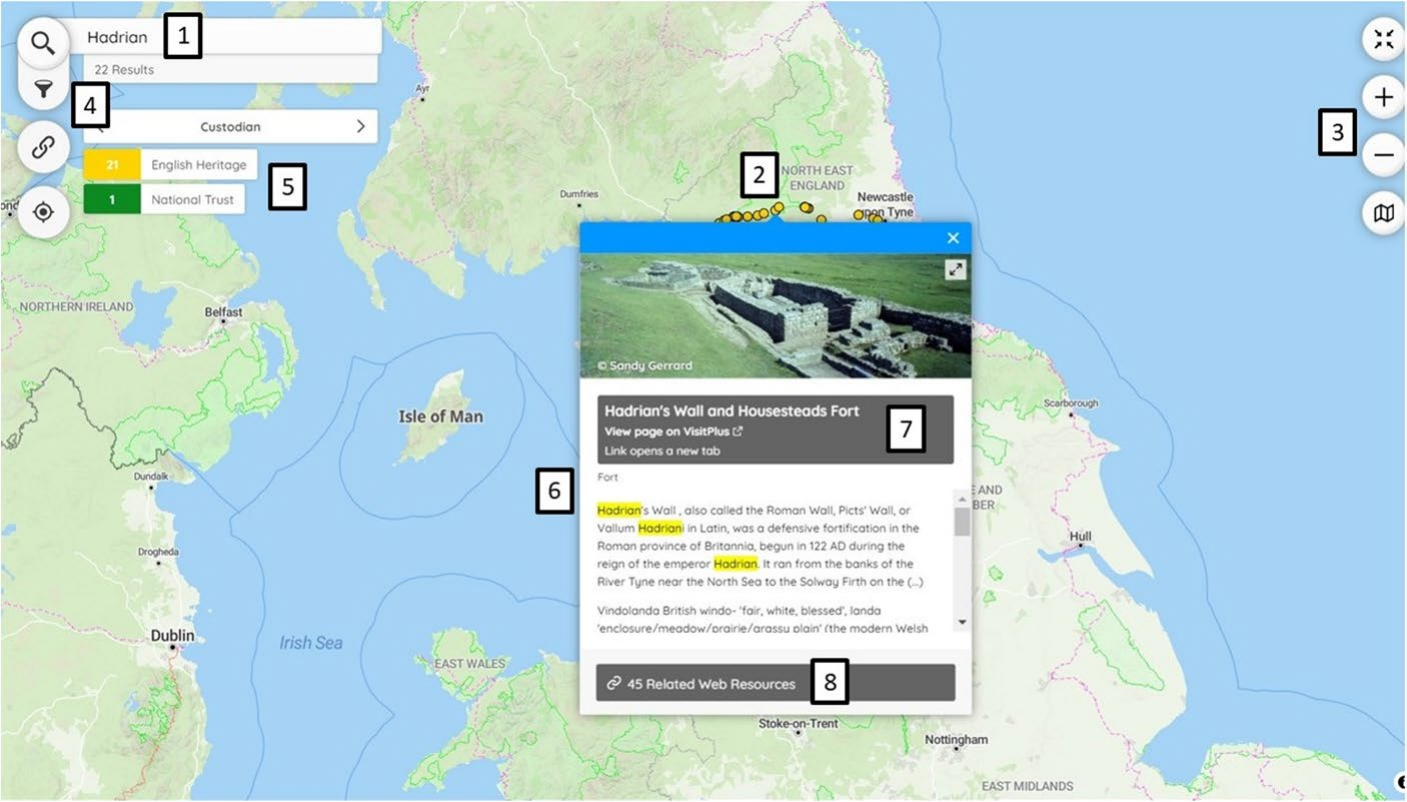
Results from searching for Hadrian in VisitPlus. 1) Search Box. 2) 22 Markers. 3) Zoom. 4) Filter Icon. 5) Filter facets. 6) Popup. 7) Primary link. 8) Related resources

These affordances were formulated by gathering requirements and informed the design of a user journey. An example of this user journey, based on the VisitPlus dataset, illustrates how these affordances were implemented in the interface (Fig. 4). On opening Visitplus, the user is greeted by the distribution of visitor sites across the UK in the form of markers. Figure 4 depicts the results of a search for ‘Hadrian’, the Roman emperor, made using the search box labelled (1). The 22 markers that are returned are distributed along Hadrian’s Wall (2). Zooming and panning the map makes it possible to explore the map and markers in more detail (3). Clicking on the filter icon (4) exposes a set of facets (5). Each facet depicts the number of sites managed by custodians, in this case English Heritage and the National Trust. Clicking on the English Heritage facet updates the map by removing associated markers. The vector markers are coloured according to the facets (2), (5). Clicking on a marker displays a popup (6) that displays a primary name and link within a prominent button (7); here, clicking on ‘Hadrian’s Wall and Housesteads Fort’ provides access to the English Heritage visitor web page. Linked open data for connected records can be accessed through the ‘Related Web Resources’ button (8) at the foot of the popup, in this case, links and names of 45 records from four cultural heritage organisations. Throughout this journey, the application conforms to WCAG 2.1AA (Web Content Accessibility Guidelines) where possible and can be used with standard assistive technologies for accessibility including Non Visual Desktop Access (NVDA) screen readers like JAWS (Job Access With Speech).

The importance of the aforementioned three affordances, user journey and eight features was established by the project team by the end of design phase and substantial changes were not made subsequently. A detailed rationale for this discovery use case is described elsewhere (Rees, 2025). The design of certain features was adapted following implementation. Vector markers, associated popups, search and filter were identified as critical to user journeys. Good design provides such features with signifiers —visual cues within the interface— to communicate relevant affordances and guide the end-user (Norman, 2013, p. 14). The approach to adding signifiers was iterative and involved implementing features according to a predetermined design, user testing these with several datasets like VisitPlus and then updating the design based on the feedback. The feedback refined interface functionality, tweaking signifiers and interaction rather than making substantial changes to the design such as adding new features. The development of the architecture and dataset were not informed by user testing.

#### Vector markers

The project team decided that the design of vector markers should be restricted to points and plain circles without provision to choose symbols or colours. See (2) in Fig. 4. This provides a simple visual overview and simplifies the underlying dataset as will be discussed later in Sect. 3.4. Different colours are automatically assigned to vector markers based on categories defined in the dataset and configuration file. Each category of data becomes a different filter facet with its own colour. The design was then supplemented by insights from user testing. Signifying the ability to click on a marker was identified as important by this testing: markers should be large enough to allow end-users to click and more complex visualisation such as heat maps obscured this ability. User testing demonstrated that providing visual signifiers of the number of records connected to markers would be a useful cue to assessing the importance of the marker. Therefore, the size of vector markers was made to vary based on the number of related resources that they represent.

#### Popups

**Fig. 5:**
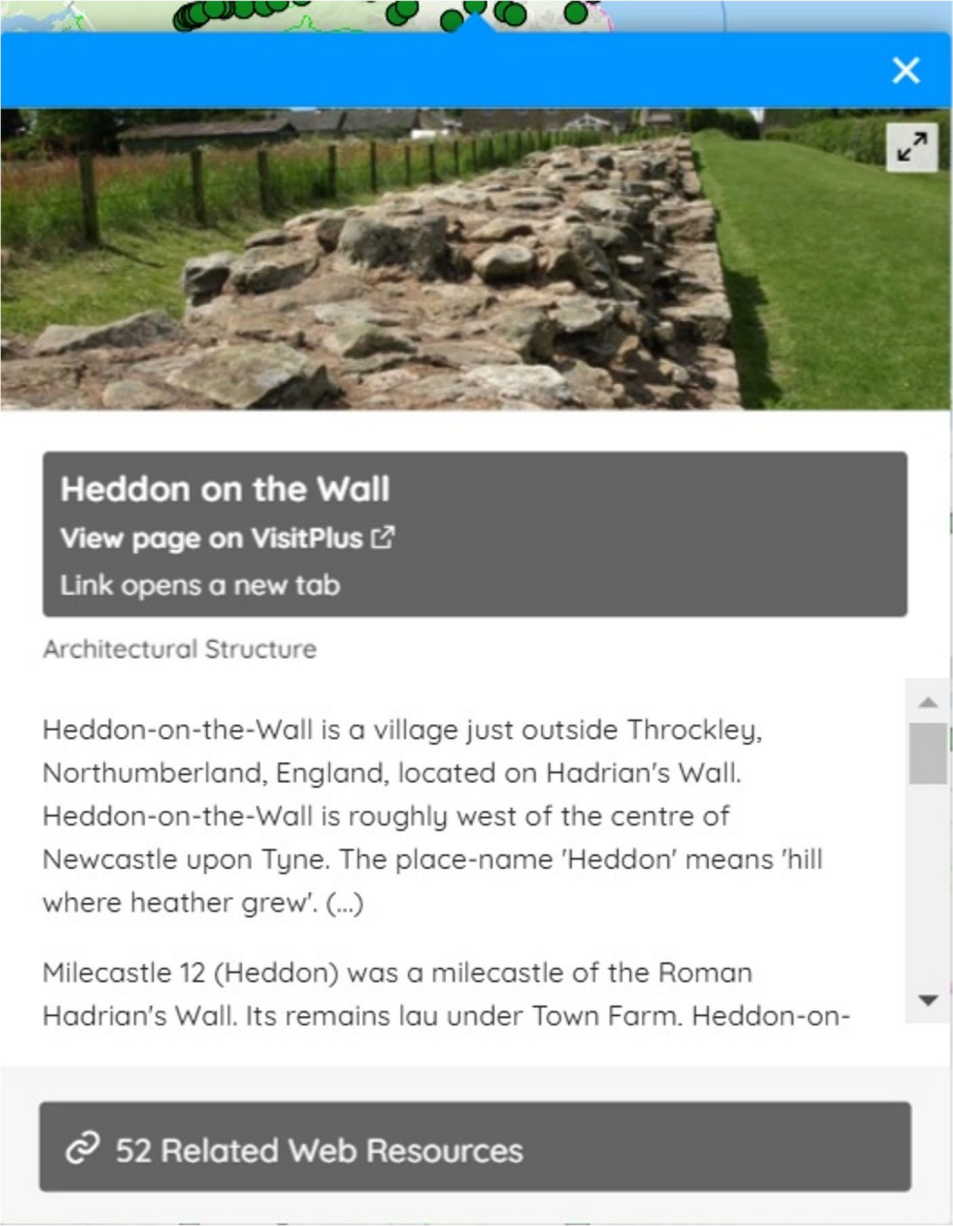
Popup in *Peripleo*

Clicking on a vector marker displays a popup with two inter-related purposes: the first to communicate the ability to click through to records and the second to display links to several connected records based on the JSON-LD (JavaScript Object Notation – Linked Data) dataset structure. For example, in VisitPlus, each popup presents not only a link to the visitor site’s web page in a main button but also links to external web pages through related resources. Each marker has one associated popup and only a single popup can be displayed on the map at any time. User testing helped us to refine the design of the popup. Primary links were made more prominent and related resources were converted from links into buttons with the addition of cultural heritage organisations’ logos. The popups were also redesigned to present information associated with records including photographs and description text, providing a preview of what the end-user might find when they click through to a record (Fig. 5).

#### Search and filter functionality

**Fig. 6:**
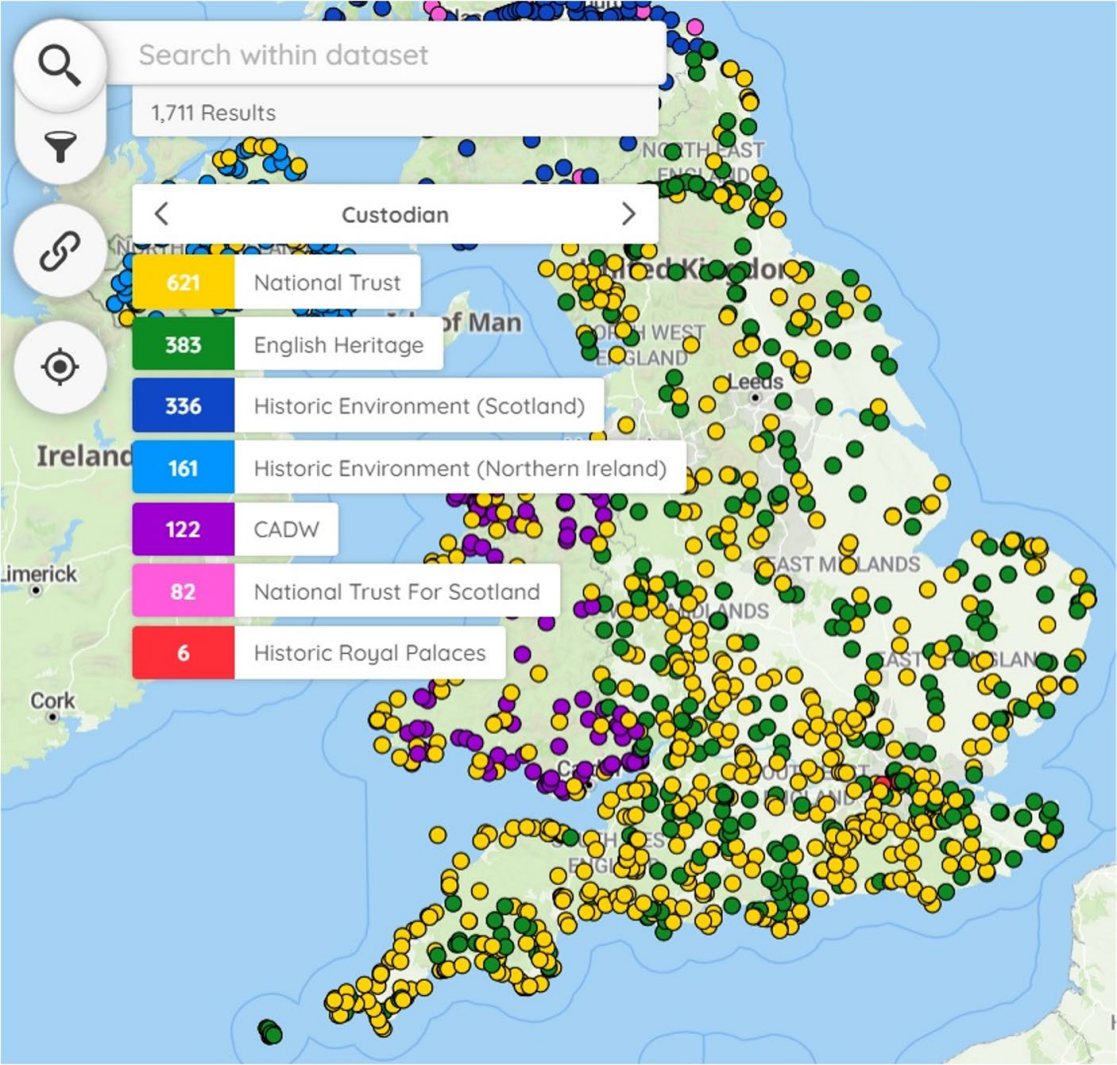
Filter used in *Peripleo*

In order to support discovery, bespoke interactive features were developed to supplement MapLibre’s core functionality, the main examples being search and filter functionality. Both reduce the number of markers on the map in order to help the end-user to discover markers of interest. Free text can be entered into the search box by the end-user. Records are displayed as markers on the map if search terms match parts of a record’s title, description or other text fields with the aim of returning the maximum number of results. A full-text index is initialised by the application automatically on application start meaning that datasets do not have to be amended by the creator. The results of searches do not update the URL and therefore cannot be shared with others. The system of filters also reduces the number of markers on the map but this time based on the end-user selecting from pre-existing facets. The filter functionality is based on a hierarchy of clickable, coloured filter facets arranged in layers (Fig. 6). The colours of the facets match vector markers helping the end-user to find a marker of interest. The creator is not provided with the option of configuring symbology or colour, a decision taken to keep the user interface, the creation of datasets and the application’s configuration simple. Coloured filter facets provide a visual summary of the collection, an overview that the end-user can drill down into based not only on preconceived queries but also on the options presented. The filters selected are stored in the URL to facilitate sharing with others. The data for these filter facets, which define displayed categories, originates from creator-provided datasets. Filter facets for a marker can be defined in spreadsheet columns before import into Locolligo. Locolligo processes these standardised terms, integrating them into the Linked Places format described below. In contrast to search, the enrichment of a dataset to populate filter facets is not always straightforward for creators as facets can be assigned to objects in several combinations. The whole process is discussed in more detail in Locolligo’s documentation (Gadd, 2023). Although Locolligo provides a graphical user interface to edit the JSON some functionality requires significant technical skill and knowledge of data structure to use.

In presenting end-users with a dataset’s breadth, *Peripleo* presents possibilities, offering the potential for end-users to discover pages that they otherwise wouldn’t find. Serendipitous discovery occurs through presenting marker options on the map but also through the display of linked open data. Bringing scattered collections together as related resources in the popup creates opportunities for serendipity, helping connected records to add up to more than the sum of their parts. These affordances can support the goals and priorities identified by LaNC’s stakeholder research. For example, end-users are motivated by geographical connections between heritage collections whilst cultural heritage organisations are interested in increasing the visibility of under-used records within collections. At the scale of the UK, the map is democratising for collections, *Peripleo* presents under-used web pages in the same visual style as their better-known counterparts. In recent years, there have been calls for interfaces to be ‘generous’. In contrast to a search box based on text, generous visual forms of interaction support varied engagement including browsing and exploration, thus catering to end-users without a specific goal. Whether *Peripleo* constitutes a ‘generous approach’ to user-interface design in Mitchell Whitelaw’s terms is unclear (Whitelaw, 2015, p. 46). Whilst *Peripleo* does ‘provide rich, navigable representations of large digital collections; they invite exploration and support browsing, using overviews to establish context and maintain orientation’, the intention was not to ‘reveal the complexity and diversity of cultural collections, and to privilege the process of interpretation.’ The latter might require more complex visualisation such as polygon geometries, symbols and relationships. Whilst these forms might motivate end-users like researchers to use *Peripleo*, such interface features could detract from organisational priorities for discovery. Furthermore, increased complexity in the design of a user interface relies on corresponding complexity in architecture and dataset. As the following sections will illustrate, the result would be problematic in terms of reusability and sustainability.

### Architecture and installation

*Peripleo’s* architecture was designed to support the complexity of the user interface whilst also being sustainable and reusable. In applying these principles the architecture was influenced by a prototype web-map application developed by Rainer Simon and Elton Barker that visualised TEI (Text Encoding Initiative) data on a map (Simon & Barker, 2022). Two parts of the architecture are the focus here: first, the hosting model and build processes chosen to make *Peripleo* available on the web and second, the configuration and installation of the application. *Peripleo* follows a client-side or front-end only design that can be supported by a static-site hosting service like Github *Pages*. This removes the need for a server and database, minimising overheads like setup, maintenance and costs. For example, the front-end model prevents the need for security updates. HTML, CSS (Cascading Style Sheets) and Javascript files can be made available on Github *Pages* free of charge and accessed through a browser. Therefore, any creator from a cultural heritage organisation with a Github account can:

1. Go to http://github.com/britishlibrary/peripleo to access the code.
2. Import the *Peripleo* repository to their own Github profile.
3. Make simple changes to the repository’s settings in Github’s graphical user interface.
4. Make their own instance of the *Peripleo* application publicly available without cost.

*Peripleo* instances made available in this way can be embedded in other websites such as blogs or websites using an HTML iframe. For example, the project embedded instances of *Peripleo* alongside explanatory text using *Simple Site* (Gadd & Rees, 2022; Padfield, 2021). This low-cost and comparatively straightforward route to hosting can expedite the application’s reuse by people working in the cultural heritage sector. Comprehensive documentation also supports these creators to make their own instances available. Of course, the chosen architecture is not without drawbacks. Sustainability is, to an extent, reliant on continuity in the provision of the Github *Pages* service. Owned by Microsoft, Github hosts the code for innumerable open-source projects, making it relatively stable. If the Github *Pages* service were to be discontinued alternatives are, however, available hosted by Gitlab, Netlify and others. A fundamental concern for reusability is that creating an instance of the application requires knowledge of Github. Although comprehensive documentation exists, the process is inevitably more complex than using platform-based applications like Google’s *MyMaps*. *Peripleo’s* documentation assumes that those creating *Peripleo* instances already host records elsewhere on the web. *Peripleo* links out to these records and does not offer the opportunity to host assets. Although it is possible to serve records on Github *Pages* in the form of web pages, this requires a separate process. Notwithstanding these issues, hosting on Github *Pages* requires the least setup and maintenance overheads of available options, making it the most sustainable and reusable method. In designing the architecture in this way, the aim was to make it as easy as possible for people working in cultural heritage organisations to become creators.

**Fig. 7:**
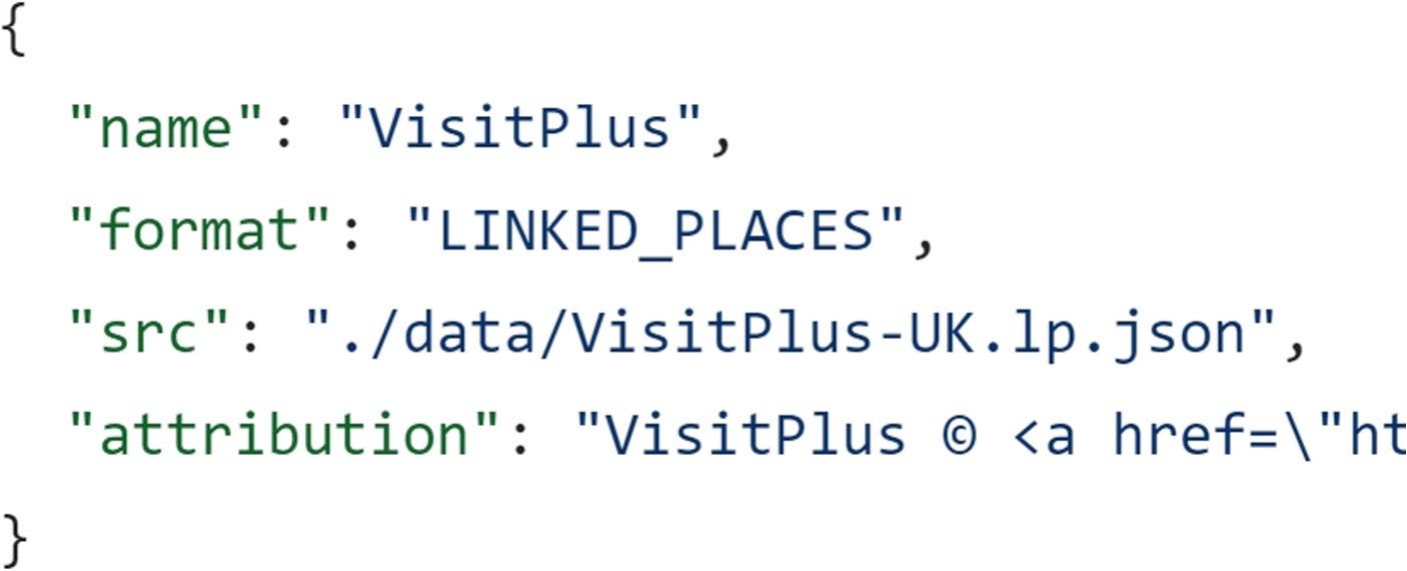
Snippet of peripleo.config.json

Client-side hosting imposes constraints that inevitably impact on the dataset and user interface. *Peripleo* was built using the React Javascript framework. This offers a component-based approach providing the ability for specialist developers, but not creators as defined here, to build new functionality. Contributions of code not only require knowledge of Javascript but also React. A JSON configuration file allows a creator setting up an instance of *Peripleo* to change aspects of layout and functionality like base layers, icons, facets, welcome message, Google *Analytics* and access to the end-user’s current location. Whilst amending this configuration file is much easier than coding new features it is still difficult for many creators working in cultural heritage to configure some options. First, familiarity with JSON is required as discussed in the next section. Second, a relatively high level of knowledge of the web and digital processes is necessary to add URLs for map tiles, *Google Analytics* codes or source icons to the configuration file. However, these changes are optional. The map can also be deployed with default settings. The only change to the configuration file that is essential to creating an instance of *Peripleo* is a simple update to the ‘src’ key in the peripleo.config.json file. This key provides the local path of the creator’s dataset or a URL for a publicly available dataset, the latter supporting the sharing of data (Fig. 7).

### Dataset

Each instance of *Peripleo* relies on a dataset to form the user interface. Datasets provide the text to populate features of the interface like filter facets and popups, alongside URLs that appear as links in the popups and coordinates that determine the position of markers on the map. The chosen Github *Pages* architecture means that datasets must be openly available and supports the sharing and reuse of datasets across instances of Peripleo. The architecture also means our datasets are based on open standards. First, the structure is based on the Open Geospatial Consortium’s GeoJSON standard used by MapLibre and major web-map components. *Peripleo* draws on a set of JSON-LD standards developed by the Pelagios Network for making historical data available. These include the Linked Places (LPF) extension to GeoJSON and Linked Traces (Kahn et al., 2021, p. 8). Stephen Gadd modified LPF that was originally developed for the exchange of gazetteer information to suit LaNC’s objectives. For each record, Title, URL and Coordinates attributes are essential whilst Description, Organisation, Type, Place name, Period, Rights, Filter facets and Relations (URLs pointing to related resources) are optional. The discussion that follows describes how the project team thought through the design of *Peripleo’s* dataset in terms of reusability.

It is possible to create an instance of Peripleo from a simple spreadsheet populated with attributes where each row represents a point on the map. This format offers the advantages of familiarity, ease of creation and opportunities for manual editing. However, spreadsheets become unmanageable when attempting to create connections between a record and several others. Therefore, the format cannot support *Peripleo’s* ability to present collections together. JSON is the most commonly used format for exchanging data on the web and provides the flexibility necessary to model linked cultural heritage data. In particular, it supports the creation of connections between a record and several others: ‘one-to-many’ relationships. This capability is crucial for accurately connecting diverse records. In order to create JSON-LD that makes the most of *Peripleo’s* functionality, creators have to bring together data from a variety of sources. For the most part data was provided by LaNC’s partner organisations in spreadsheet or tabular formats like CSV (Comma Separated Values). Such spreadsheets were not obtained in a standard way, rather, they were exported from organisations’ systems in a range of formats containing place information in varied forms such as toponyms, addresses, postcodes or coordinates. In order to create connections across these varied data, LaNC drew on a process developed by Pelagios,

‘semantic annotation encodes a link between: 1) a reference within an information-carrying resource (an online document such as a record in a numismatic database, or a toponym in a text) known as the ‘target’; and 2) entities defined by URI (Uniform Resource Identifier) in an authority file, such as a list of notable individuals or places in a geographic gazetteer, known as the ‘body’. What it does not do is create links between those entities that describe a relationship between them, such as ‘spouse of’ or ‘capital of’.’ (Vitale et al., 2021, p. 9).

**Fig. 8:**
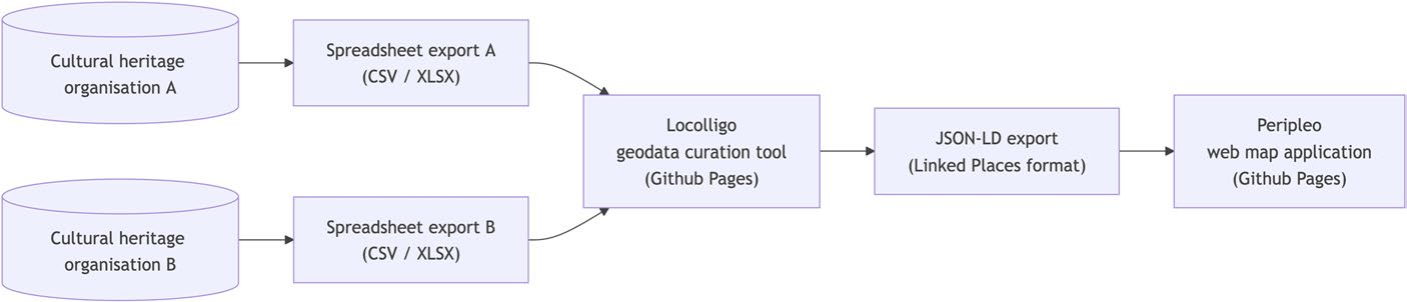
Process for creating *Peripleo* dataset

The aim is to create a dataset enriched with URIs that provide common references offering connections between records. The method has been described in detail elsewhere (Vitale et al., 2021). The focus for *Peripleo* was to use place to provide these common references in a standard way using URIs from gazetteers, authority files that also offer access to geospatial coordinates (Fig. 8).

**Fig. 9:**
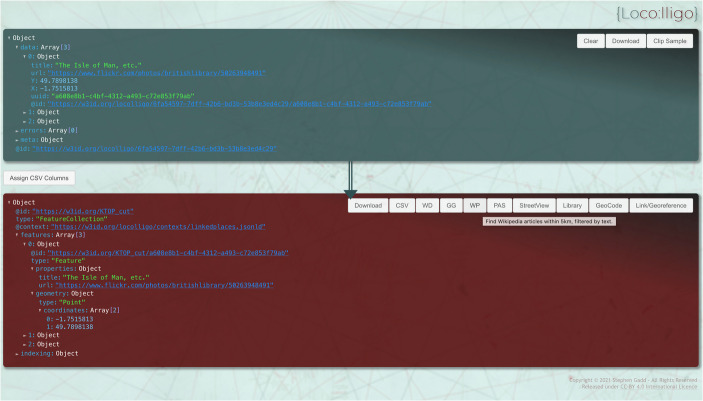
Locolligo geodata curation tool main page (Gadd, 2023)

The implementation of this approach to JSON-based formats is not straightforward, however. Editing records in JSON manually is problematic if compared to the use of spreadsheets, for example. The manual creation of ‘one-to-many’ relationships across several input datasets or the addition of filter facets is not practicable. Lowering barriers to the creation of datasets is absolutely critical to the reusability of *Peripleo*. Gadd saw a need for an intuitive and repeatable process to support creators and thus the *Locolligo* application was conceived as a prototype geodata curation tool to create datasets for *Peripleo* (Gadd, 2023). In essence it converts spreadsheets to LPF whilst offering a graphical user interface to annotate and enrich data with URIs and build connections between imported datasets as described above. Whilst freely available for external use and documented *Locolligo* still requires significant technical understanding and knowledge of data structure to use. *Locolligo* remains a prototype but was widely used by the LaNC project team and is an effective data-wrangling and curation tool in its current form (Fig. 9).

LaNC created several datasets as part of the implementation of *Peripleo* with two purposes in mind. The first was to offer insights into interface design as populating markers, popups and related resources in different ways supported user testing. The second purpose was to explore reusability through a practical understanding of the workflow required to create a dataset within *Locolligo*. The VisitPlus dataset illustrates this process. Each of the heritage visitor sites in VisitPlus is represented by coordinates and a URL for a visitor web page. Locations mentioned in GLAM records were extracted and assigned latitudes and longitudes by Victoria Morris and others. Connections were created between GLAM records and visitor sites based on geographical proximity and similarity in names using *Locolligo* and recorded in the VisitPlus dataset. Several web pages connected to each visitor location are exposed in the interface at the foot of the popup as related resources. The popup for the ‘Heddon on the Wall’ site in Fig. 5, for instance, displays records from the British Library including sounds and manuscripts as well as twenty objects found nearby recorded by the Portable Antiquities Scheme.

Above and beyond concerns regarding the JSON format, issues in data modelling require careful consideration. First, whilst geospatial data in the form of coordinates are fundamental to visualisation, they are rarely contained within GLAM records. A process by which coordinates are derived from toponyms and other geographical information is thus required. Such geographical modelling is not self-evident or straightforward and the results subjective and partial (Drucker, 2020, p. 70). GLAM records might contain several locations. Metadata for a single collection item could contain locations of origin, exchange or deposit. Other locations might exist in the record’s content including text, depiction in art or derived metrics. Such relations between record and location are ‘not to be considered certain fact, but rather’ make ‘a claim about some kind of relationship between part of the source document and the place.’ (Simon et al., 2016) It was decided not to include the type of relations in the dataset’s structure although they can be modelled within filter facets. Defining locations as coordinate geometries is fundamental to modelling data for *Peripleo*. The use of Euclidean geometry to represent place has been extensively problematised in scholarship on GIS and other technologies (Blaschke et al., 2018). The shortcomings of representing the nuance and complexity of toponyms within the simplistic demarcation of space that vector geometries provide is a concern in creating datasets for Peripleo.

Furthermore, not all of the data that LaNC dealt with bears reference to a toponym, a named place of the type included in a gazetteer. For example, the locations of archaeological finds often do not have names but are recorded as coordinates. Steps such as transforming text to coordinate geometries, connecting different records to the same place and visualising geometries on maps are subjective judgements. The intended purpose of *Peripleo* —to help the public discover pre-existing web pages through the user journey outlined earlier— influences these choices. *Peripleo’s* datasets are created to support discovery and this purpose guides modelling. For example, markers representing point geometries are sufficient for an end-user to understand a record is connected to a location of interest and open the associated popup. Similarly, communicating that a relation exists between location and record is sufficient to pique the interest of an end-user without specifying whether the relation is origin, deposition or current location. This emphasis on discovery simplifies visualisation and dataset structure thus expediting creation and enhancing reusability. In contrast to lines or polygons, point data can be represented as X/Y or Lat/Long columns in a spreadsheet format meaning *Locolligo* supports the upload of a familiar format for conversion through a simple graphical user interface.

## Discussion

### Implications for web maps in cultural heritage

*Peripleo’s* user interface and dataset structure were designed to accommodate datasets that support discovery like VisitPlus. LaNC’s exploratory technical work with other datasets underlined the difficulties in supporting other use cases within a single interface. The ‘Early Egyptian coins in Northern Europe’ dataset presents the locations of archaeological findspots of ancient base-metal and silver-alloy coins minted in Egypt, from the Hellenistic to the Byzantine periods and associated web pages (Rees et al., 2022a, p.18; Gadd & Rees, 2022). Created by Leif Isaksen with help from Gadd, the dataset drew evidence together from varied sources, including the Portable Antiquities Scheme, and published research. The dataset’s structure is akin to a GIS whereby each record, in this case the findspot of coins, is a single point marker. The implementation of the accompanying *Peripleo* instance therefore presents a geographical distribution where vector markers act as visual representations of coins. Visual analysis of patterns in the findspots could provide end-users with insights into why these Egyptian coins are found in northern Europe. However, the presentation of Early Egyptian Coins was hampered by *Peripleo’s* discovery-focused design. The simple design of vector markers, without symbols or the ability to set colours, does not offer the flexibility required to support visual analysis. Point geometries could not represent uncertainty in the locations where coins were found. Similarly, the filtering functionality and associated updates to the colours of markers stand in the way of presenting geographical patterns effectively. *Peripleo* is therefore ill-suited to presenting visual patterns that provide geographical insights. Yet these drawbacks, explored in the development and user testing phases, did not result in changes to Peripleo’s core features. Rather, the linked open data structure, simple vector markers and popup design led development to focus on supporting the presentation of several records associated with the same location effectively, as VisitPlus exemplifies.

In developing web-map interfaces, ‘all design decisions, every single one, should be made with monastic obsession toward achieving your communicative goals’ (Muehlenhaus, 2013, p. 12). *Peripleo* is not a generic web map, rather its interface supports a use case aligned with stakeholders’ requirements: discovery through the user journey and three affordances outlined earlier. Other interface designs might support use cases such as presenting spatial narratives or geographical patterns, generating research insights and encouraging open-ended browsing and exploratory behaviour. *Peripleo’s* design does not. These use cases or generous design approaches might well, however, align with the priorities of creators building web-map applications for other purposes. Alongside this focus on use case, the notion that increased complexity results in concomitant increases to labour underpinned the three principles. The discovery use case simplified dataset structure, lowering barriers to creation and hopefully encouraging reuse. *Peripleo’s* simple architecture was also chosen to support reuse. Github *Pages* provides the most sustainable architecture available although it requires knowledge to setup and leads to compromises in dataset size and format. Similarly, although linked open data supports cross-collection discovery very well, it requires specialist knowledge to work with. These drawbacks were mitigated through compromises in dataset structure and through the development of *Locolligo*. Finally, the sustainability of instances of *Peripleo* rests on its architecture. The longevity of any website relies on hosting, maintenance and updates that require expertise and financing to implement. A front-end only design where dataset and code are hosted together has attenuated the impact of these issues. Implementing principles of reusability, sustainability and discovery across the development of a single application’s architecture, dataset structure and user interface was challenging, and the need for compromise inevitable. The capabilities and motivations of the creators in cultural heritage organisations who would make instances of *Peripleo* available have been emphasised. Developing principles based on the requirements of this group has implications for supporting the adoption of software more widely and embedding this application within the practice of communities and organisations across different sectors that might not have funding or technical expertise. Moreover, the emphasis on reusability built on the Pelagios ethos of community participation where ‘different actors contribute separate but complementary ingredients’ (Kahn et al., 2021, p. 21) offering sustainability through collaboration.

### Future research and outlook

The article contributes a framework for implementing principles in application development. It outlines actionable design decisions that support adoption through a tripartite division of architecture, dataset and interface. Principles, developed through an initial phase of requirements gathering, can act as a bridge between requirements and development phases for building other applications. Ultimately, emphasising reusability and sustainability aims to inspire bottom-up empowerment and thinking beyond the platforms of multi-national tech companies. This has implications for the minimal computing approach, a paradigm that offers welcome engagement with the need for digital humanists to design under constraints. The three principles outlined here respond to the constraints that creators in cultural heritage work under, the ‘local contexts in which scholarship is being created’ (Risam & Gil, 2022, p. 4). Like many minimal computing applications, this has led *Peripleo* to adopt front-end only architecture. Furthermore, minimal computing has offered a refreshing emphasis on the practicalities of development through asking the questions:

‘1) “what do we need?”; 2) “what do we have”; 3) “what must we prioritize?”; and 4) “what are we willing to give up?”’ (Risam & Gil, 2022, p. 5).

The ‘we’ in these questions has generally emphasised those creating applications, project teams, developers or at least ‘more technical members of the DH community’ (Dombrowski, 2022, p. 5). The principles outlined in this article have tried to encompass a broader definition of ‘we’ including cultural heritage organisations and end-users. These other groups are also critical to the uptake and use of a digital application. The article’s stakeholder-based approach might be more closely aligned with the instrumentalist culture of cultural heritage organisations’ software than digital humanities research ‘tools’. Perhaps the addition of fifth and sixth questions, ‘why are we developing this application?’ and ‘for whom?’ might align minimal computing more closely with the priorities of cultural heritage. Similarly, although originally developed for scientific data management and stewardship, recent work has sought to apply the FAIR principles (Findability, Accessibility, Interoperability, Reusability) to research software. To date this work has focused on the needs of developers or research software engineers and academic researchers, emphasising the importance of metadata, standards, executability and relationships with other software (Barker et al., 2022, p. 2). The principles outlined here seek to support creators with a more diverse range of skills to create instances of *Peripleo* and make them available to the public.

*Peripleo* has been imported into over eighty repositories on Github and taken forward in different forms by Rainer Simon alongside Jamie Folsom and Performant Software. Instances have been created by the Ashmolean Museum, British Library, Cambridge University Library, Historic England, Swiss Institute of Bioinformatics, Fitzwilliam Museum, Yale University, University of Chicago and the National Library of Israel. These organisations are disparate but perhaps united by their privileged positions, with available web pages and labour to invest in new projects. Workshops delivered by the LaNC team at events such as Linked Pasts 9 and DCDC UK and specialist training for students at Jadavpur University, India and for staff at the British Library have provided instruction to help researchers and cultural heritage professionals create their own instances. Alongside technical considerations, promotion and the resulting documentation are invaluable to realising the potential for reuse outlined here (Simon et al., 2022). There is considerable scope for additional features to be added to the *Peripleo* user interface, for example, time-slider filtering, alternative projections to Web Mercator, and polygon markers. As an open-source project, *Peripleo* remains in the ‘Getting Started’ phase, with only a small group, consisting of those originally involved, supporting the application. To grow and evolve, embedding *Peripleo* within a broader range of projects and amongst a wider community is required as well as engagement with issues such as governance and resources (Arp & Forbes, 2018). Such reuse leads to sustainability as software and documentation are kept up to date amongst a community (Middle, 2023, p.367). Communities such as UK cultural heritage or Pelagios offer opportunities for positive co-dependency wherein reuse and sustainability are mutually reinforcing. This founding tenet of the Pelagios Network has resonance for software development more widely.

## Data Availability

Code and data referred to in the text: 10.23636/sd7c-k310. 10.23636/6znn-y151. 10.23636/np48-2m48. https://github.com/britishlibrary/peripleo. https://github.com/britishlibrary/peripleo-lanc.
